# Array comparative genomic hybridization in confirmation of the deleted genes in a patient with subterminal deletion of the long arm of chromosome 10 associated with sagittal craniosynostosis and dysmorphic features

**DOI:** 10.1186/1753-6561-8-S4-P1

**Published:** 2014-10-01

**Authors:** Ágatha Cristhina Faria, Ferraz de Rodrigo Toledo Atique, Maria Regina Galvêas Oliveira Rebouças, Pablo Federico Cavagnaro, Eliete Rabbi-Bortolini, Maria Rita Passos-Bueno, Flávia Imbroisi Valle Errera

**Affiliations:** 1 Universidade Federal do Espírito Santo (UFES), Vitória, Brazil; 2 Instituto de Biociências, USP, São Paulo, SP, Brazil; 3 Hospital Infantil Nossa Senhora da Glória (HINSG), Vitória, ES, Brazil; 4 Facultad de Ciencias Agrarias - Universidad Nacional de Cuyo/ INTA-EEA La Consulta, Mendoza, Argentina; 5 Associação Educacional de Vitória (AEV/FAESA), Vitória, ES, Brazil; 6 Centro de Pesquisa, EMESCAM, Vitória, ES, Brazil

## Background

Craniosynostosis results from premature ossification of one or more cranial sutures and leads to alterations in the shape of the skull and/or premature closure of cranial fontanels, causing impairment of brain perfusion, vision and hearing, airway obstruction, learning difficulties, severe cosmetic deformities and high intracranial pressure [1]. To date, the genetic mechanisms leading to sagittal craniosynostosis are poorly known. The identification of candidate genes underlying this condition may contribute to elucidation of the etiology of this common malformation. The aim of this study was to associate genotype-phenotype of a patient with a deletion in the long arm of chromosome 10 and craniosynostosis.

## Methods

A newborn female born at term (39 weeks) with birth weight 3.405 kg and length 45 cm, an occipitofrontal head circumference (OFC) of 36.5 cm, 2nd pregnancy of a mother at 39 years old and a father at 41 years old, not consanguineous and without family history of congenital anomalies was examined by neonatologists using the Mercks protocol (2003) for early identification of major and minor anomalies. GTG Banding analyzes were performed on metaphase obtained by stimulating peripheral blood lymphocytes from patient and their parents in accordance with standard procedures. The technique Multiplex ligation-dependent probe amplification (MLPA) was performed using kits for the subtelomeric regions of all chromosomes (P036 and P070-E1-B2 - MRC-Holland). Array Comparative Genome Hybridization (aCGH) (OGT CytoSure ISCA 8x60k) was performed according to the manufacturer's recommendations.

## Results and conclusions

The newborn had sagittal craniosynostosis (scaphocephaly), microcephaly, facial asymmetry, short forehead and bitemporal narrowing, microtia, hypoplastic and low-set ears, thin lips, strabismus, retromicrognathia, anteriorized anus, right single palmar crease, short neck, syndactyly of 2-3rd fingers and congenital heart disease. The karyotype was normal and MLPA detected a *de novo *deletion in region 10q26.2-q2, confirmed by aCGH that showed a 12.9 Mb deletion (122085501-135053489) (The UCSC Human Genome Project Working Draft Build 36/hg 18). Mutations in the FGFR2 gene cause several syndromes that result in craniosynostosis (Crouzon, Pfeiffer, Apert, Jackson-Weiss, Beare-Stevenson Cutis Gyrata) and familial scaphocephaly [2]. The newborn does not have any features suggestive of these syndromes, which may be due to the fact that loss of one allele results in FGFR2 haploinsufficiency instead of gain of function. The PTPRE gene (receptor tyrosine phosphatase type E) is involved in the formation and differentiation of osteoblasts and its allelic absence may explain the craniosynostosis phenotype [2]. In addition, hemizigosis for ATE1 gene may explain heart disease, as it encodes an arginyltransferase, enzyme responsible for posttranslational arginylation, a crucial molecular mechanism for the development of heart failure. Our results are corcordant with studies in mice reporting that depletion of arginyltransferase promotes congestive heart failure [3]. Other phenotypic signs found in this newborn may be related to the absence of BNIP3, MKI67, DOCK1, ADAM8 and ADAM12 genes, most of which are responsible for cell signaling, proliferation and differentiation. Reports of craniosynostosis patients with de novo deletions are important to characterize the patients phenotypes, and associate them with specific genes, which will contribute to the improvement of genetic counseling for this condition [4,5]
